# Analysis of progressive fracture in fluid‐saturated porous medium using splines

**DOI:** 10.1002/nme.7120

**Published:** 2022-09-16

**Authors:** Lin Chen, René de Borst

**Affiliations:** ^1^ Key Laboratory of Ministry of Education on Safe Mining of Deep Metal Mines Northeastern University Shenyang China; ^2^ Department of Civil and Structural Engineering University of Sheffield Sheffield UK

**Keywords:** cohesive zone model, fracture, porous medium, Powell‐Sabin B‐splines, remeshing

## Abstract

Powell‐Sabin B‐splines are employed to model progressive fracturing in a fluid‐saturated porous medium. These splines are defined on triangles and are ${𝒞}^{1}$‐continuous throughout the domain, including the crack tips, so that crack initiation can be evaluated directly at the tip. On one hand, the method captures stresses and fluid fluxes more accurately than when using standard Lagrange elements, enabling a direct assessment of the fracture criterion at the crack tip and ensuring local mass conservation. On the other hand, the method avoids limitations for discrete crack analysis which adhere to isogeometric analysis. A crack is introduced directly in the physical domain. Due to the use of triangles, remeshing and crack path tracking are straightforward. During remeshing transfer of state vectors (displacement, fluid pressure) is required from the old to the new mesh. The transfer is done using a new approach which exploits a least‐squares fit with the energy balance and conservation of mass as constraints. The versatility and accuracy to simulate free crack propagation are assessed for mode‐I and mixed‐mode fracture problems.

## 1 INTRODUCTION

Fracture in fluid‐saturated porous media happens in many applications including, for instance, petroleum and geotechnical engineering, biology and medical sciences, geothermal energy storage and so on. Modeling progressive fracturing is a challenging problem due to the complex nonlinear behavior of the solid skeleton and the fluid.

Building on the pioneering works by Terzaghi^1^ and Biot^2^ for fully coupled models of intact porous materials, several approaches have been developed for the analysis of fracture in fluid‐saturated porous media. Analytical solutions^3^, ^4^, ^5^ were derived first on the basis of simplifying assumptions, such as homogeneity and impermeability, idealized geometry and linear elasticity. The first numerical attempt to consider a discontinuity, including fluid flow in the porous medium, is by Boone and Ingraffea,^6^ who used finite differences for the flow in the crack and finite elements for the deformation of the surrounding porous medium.

Since then, a large number of numerical models have been proposed, including the extended finite element method,^7^ extended isogeometric analysis,^8^ embedded strong discontinuities,^9^ the phase‐field method,^10^, ^11^, ^12^ a coupled FEM‐peridynamics model,^13^ interface elements equipped with cohesive zones,^14^, ^15^, ^16^, ^17^ and combined finite‐discrete methods.^18^ In the extended finite element method (XFEM),^7^ the field variables (displacement and pore pressure) are approximated by a regular part and an enhanced part to incorporate the field variable jump across the crack interface.^19^ The XFEM holds unique advantages: no need to treat cracks as geometric discontinuities, and avoiding mesh refinement around crack tips. However, due to ${𝒞}^{0}$‐continuity between elements in using Lagrangian basis functions, the stress and the gradient of the pore pressure are generally discontinuous at element boundaries and the crack tip.^20^ The accuracy of the stress prediction and the pore pressure evaluation are particularly important, also in relation to the local mass conservation between elements. The extended isogeometric method (XIGA) avoids the stress and pore pressure gradient inaccuracy issue.^8^ However, to confine the influence of cracks locally, ${𝒞}^{0}$‐lines must be added in cracked elements.^21^ This leads to a reduced continuity of the basis functions, thus eliminating, or at least reducing the advantages of the isogeometric approach (namely higher order continuity). The embedded strong discontinuity method,^9^ the coupled FEM‐peridynamics model,^13^ the interface element approach^14^, ^15^, ^16^, ^17^ and combined finite‐discrete methods^18^ share same issues as in the (X)FEM: inaccurate stress and pore pressure gradient predictions. More recently, phase‐field models have been introduced to model fracturing in porous media in a smeared manner.^10^ The phase‐field approach finds its origin in the so‐called variational approach to fracture:^22^ crack initiation and propagation are considered as a minimization problem of a Griffith‐like energy functional.^11^ Currently, the phase‐field models have been only applied to the analysis of brittle fracture in porous media, not considering adhesive behavior of the crack interface.^23^ The results of phase‐field models are very sensitive to the element mesh size and the length scale. Certain assumptions are often made regarding the material parameters,^12^ such as the porosity and the Biot coefficients, but they can be somewhat arbitrary.

Within the class of discrete crack models, interface elements have gained popularity for modeling discontinuities due to their simplicity and robust performance, and have also been used to model fracture in a poroelastic medium.^14^, ^15^, ^16^, ^17^ In an analysis, the interface elements must be inserted in the mesh *a priori*, requiring a knowledge of the location of the fracture. This restricts the application of the interface elements in a general framework. Remeshing was introduced to remove this restriction, and has been used for arbitrary crack propagation in saturated porous media.^24^, ^25^

Early works on discrete crack propagation utilized Lagrange basis functions for the approximation of the field variable.^14^, ^15^ Since the stress is then discontinuous at element boundaries, including at the crack tip, the accuracy in the stress predication is lower, which is particularly important for determining the crack initiation and the prediction of the crack propagation direction. Moreover, ${𝒞}^{0}$‐continuous Lagrange bases lead to a discontinuous inter‐element pressure gradient, so that local mass conservation is not guaranteed, unless special degrees of freedom are used.^26^

Non‐uniform rational basis splines (NURBS) basis functions and T‐splines used in isogeometric analysis can remedy the stress inaccuracy and not satisfying the mass balance as well, and have been used in the framework of discrete crack simulations.^27^, ^28^ Also in this approach the enhanced continuity in the stress evaluation around the crack tip is essential for properly prediction the direction of crack propagation.^29^ However, to confine the influence of cracks, ${𝒞}^{0}$‐lines must be added.^28^ This leads to a reduced continuous basis functions, reducing the advantage of the isogeometric approach, namely higher‐order continuity. Experience also shows that when using NURBS or T‐splines the initial mesh must be sufficiently well aligned with the final crack path.^28^ This is due to the fact that crack segments are inserted in the parameter domain, and then the mesh is reparameterized in the physical domain.^28^ Meshless methods have similar issues as isogeometric analysis, such as higher‐order continuity and a wide support of cracks in the domain.^30^

In this contribution we will employ Powell‐Sabin B‐splines^31^ for progressive fracture analysis in a fluid‐saturated porous medium. Powell‐Sabin B‐splines are defined on triangles, and are ${𝒞}^{1}$‐continuous throughout the entire domain, even at crack tips. This prevents the inaccurate stress and pressure gradient evaluation when employing Lagrangian basis functions. Because of the flexibility of triangular elements, crack insertion is performed directly in the physical domain, thus avoiding the limitation adhering to isogeometric analysis. We start with a brief introduction of governing equations for the porous medium. Section 3 discuss the Powell‐Sabin finite element discretization of governing equations. In this section, the weak form, Powell‐Sabin B‐splines and poromechanical interface elements are elaborated. Next, we discuss the algorithm to insert a new crack segment, and the state vector update after crack insertions. A novel least‐square fit algorithm for remeshing is introduced here. In Section 5, numerical examples are given which demonstrate the versatility and accuracy of the method.

## 2 GOVERNING EQUATIONS FOR THE POROUS MEDIUM

**FIGURE 1:**
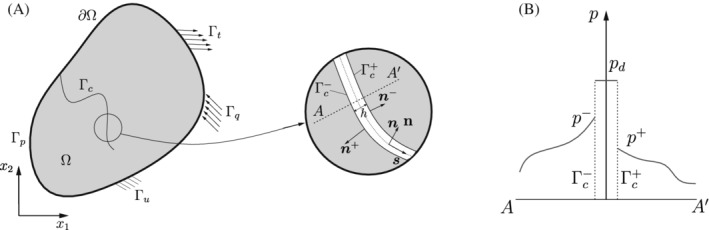
(A) A solid body Ω with an internal discontinuity $\Gamma_{c}$. $\Gamma_{c}$ is an interface boundary with positive and negative sides, $\Gamma_{c}^{+}$ and $\Gamma_{c}^{-}$, respectively. Boundary $\Gamma_{u}$ is prescribed with a displacement ū; $\Gamma_{t}$ with a prescribed traction $\hat{t}$; $\Gamma_{p}$ with a prescribed fluid pressure $\bar{p}$; $\Gamma_{q}$ with a prescribed inflow $\hat{q}$; (B) pressure around the internal discontinuity $\Gamma_{c}$

We consider a fully saturated porous medium, which consists of an elastic porous medium and a Newtonian fluid. The porous medium is split into two parts by an interface $\Gamma_{c}$ in the physical domain Ω, see Figure 1A. In this contribution, infinitesimal strains and linear elastic material behavior are assumed. There is no mass transfer or chemical interaction between the solid and the fluid. In most poroelastic systems, the deformation of the solid occur fast in comparison with the pressure change of the interstitial fluid. Thus, the solid deformation could be assumed as a quasi‐static process. Not considering gravity, convective or inertia terms, the strong form of the hydro‐static momentum equations and the boundary conditions reads:^32^

(1) $$\left\{\begin{array}{cc} \nabla \cdot \sigma =0\; & \text{on}\;\Omega ,\\ u\left(x\right)=ū\; & \text{on}\;\Gamma_{u},\\ u\left(x,\;0\right)=u_{0}\; & \text{on}\;\Omega ,\\ \dot{u}\left(x,\;0\right)={\dot{u}}_{0}\; & \text{on}\;\Omega ,\\ \sigma \cdot n=\hat{t}\; & \text{on}\;\Gamma_{t},\\ \sigma \cdot n=t_{c}\; & \text{on}\;\Gamma_{c}, \end{array}\right.$$

in which n refers to the normal vector at the boundaries. ū and $\hat{t}$ denote prescribed displacements and tractions, respectively. $u_{0}$ and ${\dot{u}}_{0}$ represent initial displacements and velocities separately. $\dot{□}$ denotes the time derivative:

(2) $$\dot{□}=\frac{\partial □}{\partial t}\;\;\;\dot{u}=\frac{\partial u}{\partial t},$$

and σ denotes the total stress defined as

(3) $$\sigma =\sigma_{s}-\alpha pI,$$

where α is the Biot coefficient, p represents the apparent fluid pressure and I denotes the unit tensor. $\sigma_{s}$ is the stress inside the solid material, which relates to the infinitesimal strain ε via

(4) $$\sigma_{s}=D:\varepsilon$$

with D the fourth‐order elastic stiffness tensor. The tractions $t_{c}$ act on the crack interface $\Gamma_{c}$, which is defined as

(5) $$t_{c}=t\left(⟦u⟧\right)-p_{d}n,$$

in which $p_{d}$ is the pressure inside the crack $\Gamma_{c}$, see Figure 1B. $t\left(⟦u⟧\right)$ are tractions due to the influence of the crack interface.

In this contribution, a cohesive‐zone model is used to model tractions across the crack. The model relates the tractions $t\left(⟦u⟧\right)$ on $\Gamma_{c}$ to the displacement jump across it. In practice, to obtain $t\left(⟦u⟧\right)$, we first compute the cohesive tractions $t_{d}$ in the local coordinate system $\left(s,\;n\right)$, see Figure 1A.

(6) $$t_{d}=t_{d}\left(⟦v⟧\right)={\left[t_{s}\;\;\;t_{n}\right]}^{T}$$

with ⟦v⟧ being the displacement jump across $\Gamma_{c}$, given in the local coordinate system $\left(s,\;n\right)$. The tractions $t\left(⟦u⟧\right)$ and the displacement jump $\left[\left[u\right]\right]$ in the global coordinate system $\left(x_{1},\;x_{2}\right)$ are then obtained via a standard transformation:

(7) $$t=R^{T}t_{d},\;\left[\left[v\right]\right]={\left[\left[\left[v_{s}\right]\right]\;\;\;\left[\left[v_{n}\right]\right]\right]}^{T}=R\left[\left[u\right]\right]=R{\left[\left[\left[u_{x_{1}}\right]\right]\;\;\;\left[\left[u_{x_{2}}\right]\right]\right]}^{T}$$

with R the rotation matrix.

In the current study, an exponential traction‐opening law is used to obtain the tractions on $\Gamma_{c}$:

(8) $$\left\{\begin{array}{l} t_{n}=t_{u}\exp\left(-\frac{t_{u}}{{𝒢}_{c}}\kappa \right)\\ t_{s}=d_{\text{int}}\exp\left(h_{s}\kappa \right)\left[\left[v_{s}\right]\right], \end{array}\right.$$

where $t_{u}$ is the fracture strength, ${𝒢}_{c}$ denotes the fracture energy, $d_{\text{int}}$ represents the initial crack shear stiffness (when κ=0), and $h_{s}=\ln\left(d_{\kappa =1.0}/d_{\text{int}}\right)$ governs the degradation of the shear stiffness. The history parameter κ is set through a loading function $f=f(\left[\left[v_{n}\right]\right],\left[\left[v_{s}\right]\right],\kappa )$, which evolves according to Kuhn–Tucker conditions.^28^

(9) $$f=\left[\left[v_{n}\right]\right]\text{or}\left[\left[v_{s}\right]\right]-\kappa ⩽0\;\dot{\kappa }⩾0\;\dot{\kappa }f=0.$$

In the case of unloading (f<0), the tractions are obtained from a secant relation. To avoid interpenetration, a penalty stiffness $k_{p}$ is specified in the normal direction.

Darcy's law is used to describe the flow of Newtonian fluids in an isotropic porous medium

(10) $$-k_{f}\nabla p=n_{f}\left(v-\dot{u}\right)$$

with v and $\dot{u}$ the velocity of the fluid and solid separately; $n_{f}$ the porosity of the medium and $k_{f}$ the effective permeability coefficient of the porous medium, $k_{f}=k/\mu$. k and μ are the intrinsic permeability of the porous medium and the viscosity of the fluid separately. Using Darcy's law, the mass balance equations of the fluid and solid phases, and boundary conditions read:^17^

(11) $$\left\{\begin{array}{ll} \alpha \nabla \cdot \dot{u}-\nabla \cdot \left(k_{f}\nabla p\right)+\frac{1}{M}\frac{\partial p}{\partial t}=0\; & \text{on}\;\Omega ,\\ p\left(x\right)=\bar{p}\; & \text{on}\;\Gamma_{p},\\ p\left(x,\;0\right)=p_{0}\; & \text{on}\;\Omega ,\\ q\cdot n=\hat{q}\; & \text{on}\;\Gamma_{q},\\ q\cdot n=q_{c}\cdot n\; & \text{on}\;\Gamma_{c}, \end{array}\right.$$

in which $\bar{p}$ and $\hat{q}$ represent the prescribed fluid pressure and inflow, respectively. $p_{0}$ denotes the initial fluid pressure. $q_{c}\cdot n$ is the inflow from the fracture, q is the fluid flux, $q=-k_{f}\nabla p$ and M represents the Biot modulus:

(12) $$\frac{1}{M}=\frac{\alpha -n_{f}}{K_{s}}+\frac{n_{f}}{K_{f}}$$

with $K_{s}$ and $K_{f}$ the solid and fluid bulk moduli, respectively.

## 3 POWELL‐SABIN FINITE ELEMENT DISCRETIZATION OF GOVERNING EQUATIONS

In this section, the governing equations for the porous medium are solved by Powell‐Sabin finite elements. We use Powell‐Sabin B‐splines for the trial functions in the solution space, and also for the parametrization of the geometry. Poromechanical interface elements are employed at the crack.

### 3.1 Weak form of the governing equations

The weak form of the balance equations (1) and (11) is obtained through multiplication by the test functions η and ζ for the solid skeleton and the interstitial pressure, respectively. Considering the internal boundaries $\Gamma_{c}^{+}$ and $\Gamma_{c}^{-}$ as well as the conditions at the external boundaries $\Gamma_{u}$, $\Gamma_{t}$, $\Gamma_{p}$, and $\Gamma_{q}$, and using the divergence theorem and integration by parts leads to the weak form:

(13a) $$\begin{array}{ll} \int_{\Omega }\nabla \eta :\left(\sigma_{s}-\alpha pI\right)d\Omega -\int_{\Gamma_{c}^{+}}\eta^{+}\cdot \left(n^{+}\cdot \sigma^{+}\right)d\Gamma -\int_{\Gamma_{c}^{-}}\eta^{-}\cdot \left(n^{-}\cdot \sigma^{-}\right)d\Gamma =\int_{\Gamma_{t}}\eta \cdot \hat{t}d\Gamma , & \end{array}$$

(13b) $$\begin{array}{ll} & \int_{\Omega }\alpha \zeta \nabla \cdot \dot{u}d\Omega +\int_{\Omega }k_{f}\nabla \zeta \cdot \nabla pd\Omega +\int_{\Omega }\frac{1}{M}\zeta \dot{p}d\Omega +\int_{\Gamma_{c}^{+}}\zeta^{+}\left(n^{+}\cdot q^{+}\right)d\Gamma \\ & \;+\int_{\Gamma_{c}^{-}}\zeta^{-}\left(n^{-}\cdot q^{-}\right)d\Gamma =-\int_{\Gamma_{q}}\zeta \hat{q}d\Gamma . \end{array}$$

The interface at the crack, $\Gamma_{c}$, introduces two terms in the weak form: cohesive tractions and the normal fluid flux through the interface faces. Taking force equilibrium conditions at both faces of the crack interface, Equation (5), we have:

(14) $$-n^{+}\cdot \sigma^{+}=n^{-}\cdot \sigma^{-}=t\left(⟦u⟧\right)-p_{d}n$$

with $n=n^{-}=-n^{+}$. We reformulate Equation (13a) using Equation (14)

(15) $$\int_{\Omega }\nabla \eta :\left(\sigma_{s}-\alpha pI\right)d\Omega +\int_{\Gamma_{c}}⟦\eta ⟧\cdot \left(t\left(⟦u⟧\right)-p_{d}n\right)d\Gamma =\int_{\Gamma_{t}}\eta \cdot \hat{t}d\Gamma$$

with $⟦\eta ⟧=\eta^{+}-\eta^{-}$.

If the pressure is discontinuous at the crack interface $\Gamma_{c}$, and using the equation $n=n^{-}=-n^{+}$, the weak form of the mass balance can be rewritten as:

(16) $$\begin{array}{ll} & \int_{\Omega }\alpha \zeta \nabla \cdot \dot{u}d\Omega +\int_{\Omega }k_{f}\nabla \zeta \cdot \nabla pd\Omega +\int_{\Omega }\frac{1}{M}\zeta \dot{p}d\Omega -\int_{\Gamma_{c}^{+}}\zeta^{+}\left(n\cdot q^{+}\right)d\Gamma \\ & \;+\int_{\Gamma_{c}^{-}}\zeta^{-}\left(n\cdot q^{-}\right)d\Gamma =-\int_{\Gamma_{q}}\zeta \hat{q}d\Gamma . \end{array}$$

### 3.2 Powell‐Sabin finite elements

**FIGURE 2:**
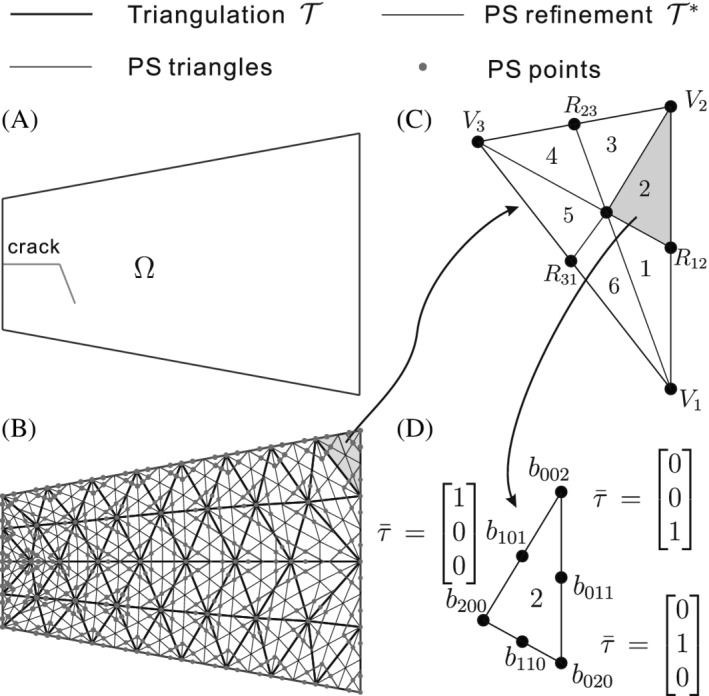
Example of a cracked domain Ω with a triangulation 𝒯 (thick black lines), Powell‐Sabin refinement ${𝒯}^{*}$ (thin black lines) of 𝒯, Powell‐Sabin triangles (red) and Powell‐Sabin points (green). In (C) each triangle e is subdivided into six mini‐triangles. In (D) each mini‐triangle has a barycentric coordinate system $\bar{\tau }$.

To discretize Equations (15) and (16), Powell‐Sabin B‐splines are employed. Powell‐Sabin B‐splines are defined on triangles, holding ${𝒞}^{1}$‐continuity throughout the entire domain, even at crack tips. Other ${𝒞}^{1}$‐continuous triangular elements have been constructed in the past, like the Argyris element,^33^ the Hsieh–Clough–Tocher (HCT) element,^34^ and natural elements.^35^ Powell‐Sabin B‐splines describe the geometry and interpolate the displacement field u and the fluid pressure p in an isoparametric sense:

(17) $$x=\overset{N_{v}}{\underset{k=1}{\sum }}\overset{3}{\underset{j=1}{\sum }}N_{k}^{j}X_{k}^{j}\;u=\overset{N_{v}}{\underset{k=1}{\sum }}\overset{3}{\underset{j=1}{\sum }}N_{k}^{j}U_{k}^{j}\;p=\overset{N_{v}}{\underset{k=1}{\sum }}\overset{3}{\underset{j=1}{\sum }}N_{k}^{j}p_{k}^{j},$$

where $X_{k}^{j}$ represent the coordinates of the corners $Q_{k}^{j}$ of the Powell‐Sabin triangles, see Figure 2B. $U_{k}^{j}$ and $p_{k}^{j}$ denote the displacement and pressure degrees of freedom (PDOFs), respectively, at $Q_{k}^{j}$, and $N_{v}$ is the total number of vertices. The indices j=1,2,3 imply that three Powell‐Sabin B‐splines are defined on each vertex k, Figure 2B.

**FIGURE 3:**
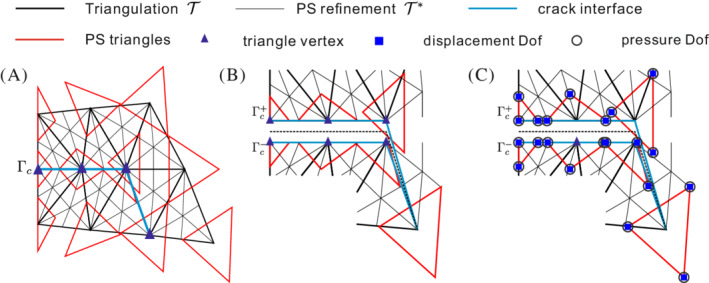
(A) Powell‐Sabin refinement ${𝒯}^{*}$ (thin black lines), Powell‐Sabin triangles (red) and triangle vertices (blue triangles) along the interface $\Gamma_{c}$; (B) enlargement of the interface $\Gamma_{c}$; (C) zero‐thickness interface elements enriched with pressure degrees of freedom (2PDOF model).

We now give a concise description of Powell‐Sabin B‐splines.^36^ We consider a cracked domain Ω (Figure 2A) with a triangulation 𝒯, Figure 2B. 𝒯 can be generated by any package for standard triangular elements, such as Gmsh.^37^ e=1,2,…,𝔼 triangles and $N_{v}$ vertices are defined on 𝒯, which is denoted by thick black lines in Figure 2B. To construct Powell‐Sabin B‐splines, each triangle e is split into six (n=1,2,…,6) mini‐triangles, compare Figure 2B. This leads to the Powell‐Sabin refinement ${𝒯}^{*}$ and Powell‐Sabin points (plotted in green in Figure 2B).^36^ A Powell‐Sabin triangle, drawn in red, is then defined for each vertex k.^38^ We constrain the Powell‐Sabin triangles on the boundary as follows: (i) for an angle $\gamma <180^{\circ }$, two sides of the Powell‐Sabin triangle must be aligned with the two boundary edges, (ii) for an angle $\gamma =180^{\circ }$, one side of the Powell‐Sabin triangle must be aligned with the boundary edge. Powell‐Sabin triangles are not restricted by these constrains along an internal discontinuity (crack interface), see Figure 3B.

Each mini‐triangle n of element e has a barycentric coordinate system $\bar{\tau }={\left[\begin{array}{ccc} {\bar{\tau }}_{1} & {\bar{\tau }}_{2} & {\bar{\tau }}_{3} \end{array}\right]}^{T}$ and Bézier ordinates $b_{r,s,t}$, compare Figure 2C,D. Each vertex k with coordinate $V_{k}=\left(x_{1}^{k},x_{2}^{k}\right)$ has three Powell‐Sabin B‐splines $N_{k}^{j}$, j=1,2,3, that is, one for each corner $Q_{k}^{j}$ of the Powell‐Sabin triangle of vertex k. The coordinate of $Q_{k}^{j}$ is defined as $\left(x_{1}^{k,j},x_{2}^{k,j}\right)$.^38^ The Powell‐Sabin B‐splines $N_{k}^{j}$ over each mini‐triangle, Figure 2D, can be obtained using the Bézier ordinates $b_{r,s,t}$:^38^

(18) $$N_{k}^{j}\left(\bar{\tau }\right)=\underset{r+s+t=2}{\sum }b_{r,s,t}B_{r,s,t}^{2}\left(\bar{\tau }\right)\;\text{with}\;B_{r,s,t}^{2}\left(\bar{\tau }\right)=\frac{2!}{r!s!t!}{\bar{\tau }}_{1}^{r}{\bar{\tau }}_{2}^{s}{\bar{\tau }}_{3}^{t},$$

where $B_{r,s,t}^{2}\left(\bar{\tau }\right)$ represent the Bernstein polynomials.

The Bézier ordinates $b_{r,s,t}$ are obtained by considering the properties of Powell‐Sabin B‐splines at each vertex k.^36^ We can implement the Powell‐Sabin B‐splines in existing finite element codes by Bézier extraction

(19) $$N_{n}^{e}=C_{n}^{e}B$$

with $C_{n}^{e}$ a matrix filled by Bézier ordinates, $N_{n}^{e}$ Powell‐Sabin B‐splines associated with each mini‐triangle n in element e, and B Bernstein polynomials.

Powell‐Sabin B‐splines do not satisfy the Kronecker‐delta property and are non‐interpolatory at the vertex.^39^ Thus, imposing essential boundary conditions on $\Gamma_{u}$ and $\Gamma_{p}$ is not as trivial as in standard finite elements. In this contribution, we will employ Lagrange multipliers to weakly impose essential boundary conditions. The Lagrange multiplier method introduces a new unknown vector λ. We discretize the Lagrange multipliers λ and the field variables ψ, that is, the displacement u and the fluid pressure p on essential boundaries by using Powell‐Sabin B‐splines:

(20) $$\lambda =\overset{N_{bv}}{\underset{k=1}{\sum }}\overset{3}{\underset{j=1}{\sum }}N_{k}^{j}\lambda_{k}^{j}=N_{b}^{e}\lambda_{b}^{e},\;\psi =\overset{N_{bv}}{\underset{k=1}{\sum }}\overset{3}{\underset{j=1}{\sum }}N_{k}^{j}\Psi_{k}^{j}=N_{b}^{e}\Psi_{b}^{e},$$

with $N_{bv}$ total number of vertices on the essential boundary $\Gamma_{u}$. $N_{b}^{e}$ denotes the element boundary shape function matrix. $\lambda_{b}^{e}$ and $\Psi_{b}^{e}$ denote degrees of freedom associated with element boundaries.

We take the enforcement of the displacement boundary condition u=ū as an illustration. Considering ψ=u in Equation (20), and subsequently applying the Lagrange multiplier method, Equation (15) can be reformulated as^39^

(21) $$\begin{array}{ll} & \int_{\Omega }\nabla \eta :\left(\sigma_{s}-\alpha pI\right)d\Omega +\int_{\Gamma_{c}}⟦\eta ⟧\cdot \left(t\left(⟦u⟧\right)-p_{d}n\right)d\Gamma \\ & \;-\int_{\Gamma_{t}}\eta \cdot \hat{t}d\Gamma -\int_{\Gamma_{u}}\lambda \cdot \left(u-ū\right)\;d\Gamma =0. \end{array}$$

Lagrange multipliers λ introduce an additional unknown on the boundary $\Gamma_{u}$, which can be interpreted as a reactive traction, that is, $\lambda =\sigma \left(u\right)\cdot n$ on $\Gamma_{u}$. Linearizing Equation (21) results in the corresponding system of equations.

### 3.3 Poromechanical interface elements in the Powell‐Sabin finite element scheme

The fluid inside the crack $\Gamma_{c}$ will induce pressures on the crack faces. To consider this effect we enhance the interface elements with porosity. The standard interface element is augmented with PDOFs. Since Powell‐Sabin B‐splines do not satisfy the Kronecker‐delta property and are non‐interpolatory at the vertex, the augmentation with pressure DOFs is not as standard as when using Lagrange basis functions. We will consider the model of two pressure degrees of freedom (2PDOF) as an example. For a 2PDOF model 2PDOF are added, one on each side of the crack, which allows a discontinuous pressure across the crack $\Gamma_{c}$.

In the 2PDOF model the pressures at each side of the crack are independent, which allows for a discontinuous pressure across the interface element, see Figure 3C. Analogous to Darcy's law, the fluid transport across the crack interface is then given as^32^

(22) $$n\cdot q^{-}=n\cdot q^{+}=n\cdot q=-k_{i}\left(p^{+}-p^{-}\right)=-k_{i}\left(h_{p}^{+}p^{+}-h_{p}^{-}p^{-}\right)=-k_{i}H_{p}\tilde{p}$$

with $k_{i}$ the interface permeability, $H_{p}$ is the pressure jump matrix, $H_{p}=\left[h_{p}^{+}\;\;\;-h_{p}^{-}\right]$; $h_{p}^{+}$ and $h_{p}^{-}$ are the shape function matrices associated with the crack interfaces $\Gamma_{c}^{+}$ and $\Gamma_{c}^{-}$, respectively. The arrays $p^{+}$ and $p^{-}$ contain the PDOFs at both sides of the interface, and $\tilde{p}={\left[{\left(p^{+}\right)}^{T}\;\;\;{\left(p^{-}\right)}^{T}\right]}^{T}$.

In 2PDOF model there is no independent fluid pressure within the crack. Consequently, the pressure term vanishes in the traction continuity condition Equation (5), and Equation (23) is rewritten as:

(23) $$\int_{\Omega }\nabla \eta :\left(\sigma_{s}-\alpha pI\right)d\Omega +\int_{\Gamma_{c}}⟦\eta ⟧\cdot \left(t\left(⟦u⟧\right)\right)d\Gamma =\int_{\Gamma_{t}}\eta \cdot \hat{t}d\Gamma .$$

Substituting the interface term, Equation (22), into the weak form of the mass balance, Equation (16), leads to:

(24) $$\begin{array}{ll} & \int_{\Omega }\alpha \zeta \nabla \cdot \dot{u}d\Omega +\int_{\Omega }k_{f}\nabla \zeta \cdot \nabla pd\Omega +\int_{\Omega }\frac{1}{M}\zeta \dot{p}d\Omega +\int_{\Gamma_{c}^{+}}\zeta^{+}k_{i}H_{p}\tilde{p}d\Gamma \\ & \;-\int_{\Gamma_{c}^{-}}\zeta^{-}k_{i}H_{p}\tilde{p}d\Gamma =-\int_{\Gamma_{q}}\zeta \hat{q}d\Gamma . \end{array}$$

The mass conservation Equation (24) contains time derivatives, which are discretized using the Backward Euler scheme:

(25) $$\dot{□}=\frac{{□}^{t+\Delta t}-{□}^{t}}{\Delta t}.$$

In combination with the Powell‐Sabin approximation Equation (17) the weak form, Equations (23) and (24), yields:

(26a) $$\int_{\Omega }B^{T}\sigma_{s}d\Omega -\int_{\Omega }\alpha B^{T}mN_{p}p^{t+\Delta t}d\Omega +\int_{\Gamma_{c}}H^{T}t\left(⟦u⟧\right)d\Gamma =\int_{\Gamma_{t}}N^{T}\hat{t}d\Gamma ,$$

(26b) $$\begin{array}{ll} & \int_{\Omega }\alpha N_{p}^{T}m^{T}B\left(U^{t}-U^{t+\Delta t}\right)d\Omega +\int_{\Omega }\frac{1}{M}N_{p}^{T}N_{p}\left(p^{t}-p^{t+\Delta t}\right)d\Omega \\ & \;-\Delta t\int_{\Omega }k_{f}B_{p}^{T}B_{p}p^{t+\Delta t}d\Omega -\Delta t\int_{\Gamma_{c}}k_{i}H_{p}^{T}H_{p}{\tilde{p}}^{t+\Delta t}d\Gamma =\Delta t\int_{\Gamma_{p}}N_{p}^{T}\hat{q}d\Gamma , \end{array}$$

where $m={\left[1\;\;1\;\;0\right]}^{T}$. Matrices N, B, and H are related to the displacement degree of freedom U, and contain shape functions, their derivatives, and relative displacements, respectively.^38^ $N_{p}$ and $B_{p}$ are shape functions and their derivatives related to the pressure degree of freedom p.

Linearization of Equation (26) leads to equations for the Newton–Raphson iterative scheme:

(27) $$\left[\begin{array}{cc} K_{\text{uu}}^{\Omega }+K_{\text{uu}}^{\Gamma_{c}} & K_{\text{up}}^{\Omega }\\ K_{\text{pu}}^{\Omega } & M_{\text{pp}}^{\Omega }+K_{\text{pp}}^{\Omega }+K_{\text{pp}}^{\Gamma_{c}} \end{array}\right]\left[\begin{array}{c} \Delta U\\ \Delta p \end{array}\right]=F_{\text{ext}}-F_{\text{int}}$$

with $F_{\text{ext}}$ and $F_{\text{int}}$ being external and internal force vectors, which are obtained from Equation (26). The stiffness matrices are defined as:

$$\begin{array}{llll} & K_{\text{uu}}^{\Omega }=\int_{\Omega }B^{T}DBd\Omega \;\;\;K_{\text{uu}}^{\Gamma_{c}}=\int_{\Gamma_{c}}H^{T}R^{T}T_{d}RHd\Gamma \; & & \\ & K_{\text{up}}^{\Omega }=-\int_{\Omega }\alpha B^{T}mN_{p}d\Omega \;\;K_{\text{pu}}^{\Omega }={\left(K_{\text{up}}^{\Omega }\right)}^{T}\; & & \\ & M_{\text{pp}}^{\Omega }=-\int_{\Omega }\frac{1}{M}N_{p}^{T}N_{p}d\Omega \;\;K_{\text{pp}}^{\Omega }=-\Delta t\int_{\Omega }k_{f}B_{p}^{T}B_{p}d\Omega \; & & \\ & K_{\text{pp}}^{\Gamma_{c}}=-\Delta t\int_{\Gamma_{c}}k_{i}H_{p}^{T}H_{p}d\Gamma \; & & \end{array}$$

with R the rotation matrix and $T_{d}$ the tangent stiffness of traction‐opening law at the interface $\Gamma_{c}$.^38^

## 4 ADAPTIVE ANALYSIS FOR CRACK GROWTH

We now present the algorithm to insert a new crack segment, including remeshing after a crack insertion. Then, we will discuss how to map state vectors, displacement and fluid pressure, onto the new mesh. The mapping is performed under constraints from the energy balance and mass conservation.

### 4.1 Crack insertion and domain remeshing

**FIGURE 4:**
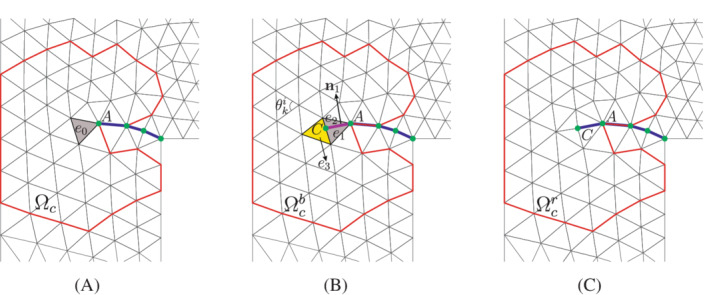
An example of crack insertion in the case of crack propagation. The blue solid curve denotes the crack interface $\Gamma_{c}$. Point A denotes the old crack tip, while point C is the new crack tip. Segment AC represents the new crack interface. $\Omega_{c}$ is the remeshing domain, confined in the red polygon. $\Omega_{c}^{b}$ and $\Omega_{c}^{r}$ are the mesh before and after remeshing $\Omega_{c}$. (A) Old mesh; (B) crack insertion; (C) domain remesh (new mesh)

Considering the ${𝒞}^{1}$‐continuity of Powell‐Sabin B‐splines at the crack tip, point A in Figure 4A, the crack initiation can be evaluated directly at this point by comparing the major principal stress $\sigma_{1}$ and the tensile strength $t_{u}$. When $\sigma_{1}⩾t_{u}$, a crack is inserted through the entire element $e_{0}$ in front of the crack tip, see Figure 4B. The new crack tip is now at point C. Due to lack of information about the possible curvature of the crack, it is introduced as a straight line within the element.^38^ The normal vector $n_{1}$ of the new crack, line AC in Figure 4B, can be obtained directly from the stress tensor at the crack tip due to the ${𝒞}^{1}$‐nature of Powell‐Sabin B‐splines. However, in this study, to further improve the quality of the prediction of the crack propagation direction, we employ an average stress tensor over a small finite domain to compute $n_{1}$. The averaging procedure is performed using a Gaussian weight function:^40^

(28) $$w=\frac{1}{{\left(2\pi \right)}^{\frac{3}{2}}l^{3}}\exp\left(-\frac{r^{2}}{2l^{2}}\right)$$

with r the distance to the crack tip, and l a smoothing length, typically taken around three times a representative element size. The crack insertion strategy bears resemblance to the “rotation of edges” strategy,^41^ but the remeshing now ensures a proper aspect ratio of the elements.

After inserting a new crack segment, element $e_{0}$ is split into two triangles $e_{1}$ and $e_{2}$, Figure 4B. Consequently, the element next to the new crack tip, $e_{3}$ in Figure 4B, has four vertices, which is not possible for the definition of Powell‐Sabin B‐splines. Thus we need to remesh the domain $\Omega_{c}$ with the new crack tip, see Figure 4C. Vertices on and outside the domain $\Omega_{c}$ and crack tips will not move. In determining the domain $\Omega_{c}$, we stand at the element with the newly inserted crack segment, shaded grey in Figure 4A. Then, a radial marching is done until three elements have been crossed in all directions, see Figure 4. The elements along one side of the crack interface are excluded, which avoids updating the field variables along the crack interface.

Remeshing then proceeds by first adjusting the elements in order to avoid triangles with four vertices and a bad aspect ratio, subsequently by fixing a polygon $\Omega_{c}$ with previously adjusted elements, and finally by solving an optimization problem of the interior angles θ of each triangle:

(29) $$\begin{array}{ll} \max\; & \;\theta_{\min}^{j}\;\;\left(j=1,\;2,\ldots \right)\\ \text{subject to:} & \;\theta_{k}^{i}\geq \theta_{\min}^{j}\;\;\theta_{\min}^{j}\geq \pi /6, \end{array}$$

where $\theta_{k}^{i}$ is the ith interior angle (i=1,2,3) of triangle k. The remeshing procedure proceeds sequentially: we first obtain the minimum interior angle $\theta_{\min}^{1}$ by solving Equation (29), then we further remesh the domain by using Equation (29) to maximize the second minimum interior angle $\theta_{\min}^{2}$ of all triangles inside $\Omega_{c}$. We repeat the procedure until all interior angles have attained a maximum value.

### 4.2 State vector update after crack insertion

New elements and vertices are introduced after the insertion of a new crack segment. Remeshing $\Omega_{c}$ is necessary to ensure elements with a suitable aspect ratio, which yields a modification of the mesh.^38^ Consequently, Powell‐Sabin B‐spline functions must be computed on new triangles. Here, the mesh before remeshing is denoted as $\Omega_{c}^{b}$, while the mesh after remeshing is represented as $\Omega_{c}^{r}$. For non‐linear problems, remeshing also requires a transfer or mapping of state vector, displacement and fluid pressure, from the old mesh $\Omega_{c}^{b}$ to the new mesh $\Omega_{c}^{r}$ at time step t and t+Δt, respectively. Next, we take the transfer of state vector at time step t+Δt as an illustrative case.

We first map the displacement ${}^{t+\Delta t}U$ from the old mesh $\Omega_{c}^{b}$ onto the new mesh $\Omega_{c}^{r}$. We define $N_{b}^{u}$ $\left(N_{b}^{p}\right)$ and $N_{r}^{u}$ $\left(N_{r}^{p}\right)$ as the displacement (fluid pressure) shape function matrix associated with the old mesh $\Omega_{c}^{b}$ and the new mesh $\Omega_{c}^{r}$, respectively. Furthermore, $U_{b}$ $\left(u_{b}\right)$ and $U_{r}$ $\left(u_{r}\right)$ are displacements related to $\Omega_{c}^{b}$ and $\Omega_{c}^{r}$, respectively. Due to the non‐interpolatory property of Powell‐Sabin B‐splines a least‐square fit is employed to carry out the mapping:

(30) $$\psi =\int_{\Omega_{c}}\left\|{}^{t+\Delta t}u_{b}-{}^{t+\Delta t}u_{r}\right\|d\Omega =\int_{\Omega_{c}}\left\|{}^{t+\Delta t}N_{b}^{u}\;\;{}^{t+\Delta t}U_{b}-{}^{t+\Delta t}N_{r}^{u}\;\;{}^{t+\Delta t}U_{r}\right\|d\Omega .$$

In general least‐square fit according to Equation (30) does not guarantee conservation of energy during the mapping from mesh $\Omega_{c}^{b}$ to mesh $\Omega_{c}^{r}$.^42^ To minimize the energy difference between $\Omega_{c}^{b}$ and $\Omega_{c}^{r}$, Equation (30) is solved by matching the energies related to the meshes $\Omega_{c}^{b}$ and $\Omega_{c}^{r}$, which is given as

(31) $$\underset{W_{\text{int}}^{b}}{\underbrace{\int_{\Omega_{c}^{b}}\varepsilon :\sigma_{s}d\Omega }}+\underset{W_{\text{coh}}^{b}}{\underbrace{\int_{\Gamma_{c}^{b}}⟦u⟧\cdot \left(t⟦u⟧\right)d\Gamma }}=\underset{W_{\text{int}}^{r}}{\underbrace{\int_{\Omega_{c}^{r}}\varepsilon :\sigma_{s}d\Omega }}+\underset{W_{\text{coh}}^{r}}{\underbrace{\int_{\Gamma_{c}^{r}}⟦u⟧\cdot \left(t⟦u⟧\right)d\Gamma }},$$

where only the internal work $W_{\text{int}}$ and the work $W_{\text{coh}}$ related to the cohesive traction on the crack surface are considered, due to their links with the displacement u.

For fracture propagation in fluid‐saturated media, we also need to transfer the fluid pressure from the old mesh $\Omega_{c}^{b}$ to the new mesh $\Omega_{c}^{r}$. This is done in a similar way to that in Equation (30):

(32) $$\chi =\int_{\Omega_{c}}\left\|{}^{t+\Delta t}p_{b}-{}^{t+\Delta t}p_{r}\right\|d\Omega =\int_{\Omega_{c}}\left\|{}^{t+\Delta t}N_{b}^{p}\;\;{}^{t+\Delta t}p_{b}-{}^{t+\Delta t}N_{r}^{p}\;\;{}^{t+\Delta t}p_{r}\right\|d\Omega .$$

Equation (32) does not guarantee conservation of mass between the mesh $\Omega_{c}^{b}$ and $\Omega_{c}^{r}$. To minimize the difference, Equation (32) is optimized by conserving the mass related to $\Omega_{c}^{b}$ and $\Omega_{c}^{r}$:

(33) $$\begin{array}{ll} & \int_{\Omega_{c}^{b}}k_{f}\nabla p\cdot \nabla pd\Omega -\int_{\Gamma_{c}^{b+}}p^{+}\left(n\cdot q^{+}\right)d\Gamma +\int_{\Gamma_{c}^{b-}}p^{-}\left(n\cdot q^{-}\right)d\Gamma =\int_{\Omega_{c}^{r}}k_{f}\nabla p\cdot \nabla pd\Omega \\ & \;-\int_{\Gamma_{c}^{r+}}p^{+}\left(n\cdot q^{+}\right)d\Gamma +\int_{\Gamma_{c}^{r-}}p^{-}\left(n\cdot q^{-}\right)d\Gamma , \end{array}$$

where only mass terms related to the pressure p are considered. In solving Equations (30) and (32) we have to consider Dirichlet boundary conditions:

(34) $$u=\hat{u}\;\;\text{on}\;\Gamma_{\Omega_{c}}^{u},\;\;p=\bar{p}\;\;\text{on}\;\Gamma_{\Omega_{c}}^{p},$$

where $\Gamma_{c}^{u}$ and $\Gamma_{c}^{p}$ are boundaries with prescribed displacement and fluid pressure, respectively. Here we fix the degree of freedom on the red polygonal boundary, see Figure 4C for instance, and along the crack path $\Gamma_{c}$.

In sum, to map the displacement and the fluid pressure from the old mesh $\Omega_{c}^{b}$ to the new mesh $\Omega_{c}^{r}$ we must solve the following optimization problems with boundary condition Equation (34):

(35a) $$\begin{array}{ll} & \min\;\;\;\;\;\int_{\Omega_{c}}\left\|{}^{t+\Delta t}N_{b}^{u}\;\;{}^{t+\Delta t}U_{b}-{}^{t+\Delta t}N_{r}^{u}\;\;{}^{t+\Delta }U_{r}\right\|d\Omega \;\;\;\;\;\;\\ & \text{subject to:}\;\;W_{\text{int}}^{b}+W_{\text{coh}}^{b}-W_{\text{int}}^{r}-W_{\text{coh}}^{r}=0\;\;\;\text{on}\;\Omega_{c}^{b}\;\text{and}\;\Omega_{c}^{r}\\ & \;\;\;\;\;\;\;\;\;\;u=\hat{u}\;\;\;\;\;\;\;\text{on}\;\Gamma_{\Omega_{c}}^{u}, \end{array}$$

and

(35b) $$\begin{array}{ll} & \min\;\;\;\;\;\int_{\Omega_{c}}\left\|{}^{t+\Delta t}N_{b}^{p}\;\;{}^{t+\Delta t}p_{b}-{}^{t+\Delta t}N_{r}^{p}\;\;{}^{t+\Delta t}p_{r}\right\|d\Omega \;\;\;\;\;\;\\ & \text{subject to:}\;\int_{\Omega_{c}^{b}}k_{f}\nabla p\cdot \nabla pd\Omega -\int_{\Gamma_{c}^{b+}}p^{+}\left(n\cdot q^{+}\right)d\Gamma +\int_{\Gamma_{c}^{b-}}p^{-}\left(n\cdot q^{-}\right)d\Gamma \\ & \;\;=\int_{\Omega_{c}^{r}}k_{f}\nabla p\cdot \nabla pd\Omega -\int_{\Gamma_{c}^{r+}}p^{+}\left(n\cdot q^{+}\right)d\Gamma +\int_{\Gamma_{c}^{r-}}p^{-}\left(n\cdot q^{-}\right)d\Gamma \\ & \;\;\;\;\;\;\;\;\;\;p=\bar{p}\;\;\;\;\;\;\;\text{on}\;\Gamma_{\Omega_{c}}^{p}. \end{array}$$

Herein, the MATLAB function *fmincon* has been used to find the optimum in Equation (35). In general, the constraint equations from the energy balance and mass conservation reduce the error of the state vector update. An error analysis of the state vector update with constraint equations has been given in Reference 42.

The computational efficiency of the proposed method is lower than that in standard FEM. In the evaluation of Equation (35) we need to find the state vector of Gauss points on the refined mesh from the old mesh.^43^ For each triangular element there are six mini‐triangles used to perform the integration. Thus, the number of triangles used in the integration is $N_{e}\times 6$, and the number of Gauss points on the refined mesh is $N_{g}\times N_{e}\times 6$, where $N_{e}$ denotes the total number of triangular elements and $N_{g}$ is the number of Gauss integration points inside each mini‐triangle. In standard FEM the number of Gauss points is $N_{g}\times N_{e}$, which is smaller than that in the proposed method. Thus, the computation time in the proposed method is increased compared to standard FEM.

## 5 NUMERICAL EXAMPLES

Below we will consider three examples. The first example deals with a pre‐fractured specimen (stationary crack), in order to benchmark the proposed method. The last two examples feature crack propagation under pure mode‐I and mixed‐mode loading conditions, demonstrating the ability of the method to analyze the propagation of cracks.

### 5.1 Stationary crack: Square plate with a center crack

**FIGURE 5:**
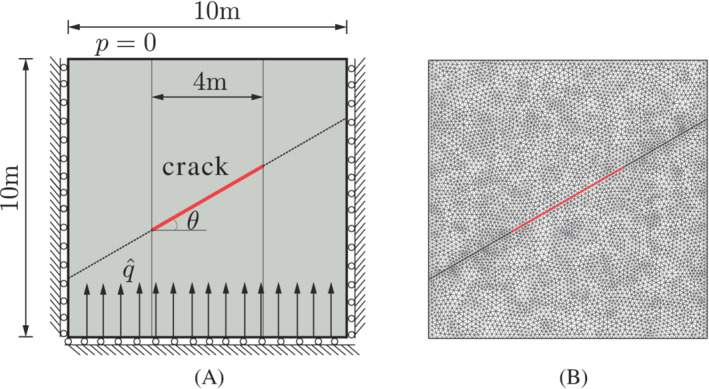
Square plate with a center crack. The crack is introduced along the red line. (A) Geometry and boundary conditions; (B) initial triangulation

First, a square plate with an inclined center crack is considered, see Figure 5. The tilt angle of the crack is chosen as θ=π/6. A constant flux $\hat{q}=10^{-4}\;m/s$ is imposed at the bottom of the plate. All boundaries are impermeable except for the top, where the fluid is allowed to flow freely. Furthermore, the bottom edge can move freely in the horizontal direction, and the right and left edges can move freely in the vertical direction. A plane‐strain condition is assumed and the crack faces are assumed to be traction‐free. The material properties of the solid are given as: Young's modulus E=9GPa, Poisson's ratio ν=0.4, Biot modulus $M=10^{18}\;\text{MPa}$, Biot coefficient α=1, porosity $n_{f}=0.3$ and the intrinsic permeability of the porous medium $k=10^{-12}\;m^{2}$. The interface is assumed to be impermeable, rendering the interface permeability $k_{i}=0$. The fluid viscosity is taken as $\mu =10^{-9}\;\text{MPa s}$. The plate has been discretized as shown in Figure 5B. A time step size Δt=1s is used until a steady state is reached at t=40s. Dirichlet boundary conditions are enforced by Lagrange multipliers as described in Section 3.2. To avoid interpenetration, a penalty stiffness $k_{p}=10^{10}\;\text{MPa/mm}$ is specified in the normal direction of the crack opening.

**FIGURE 6:**
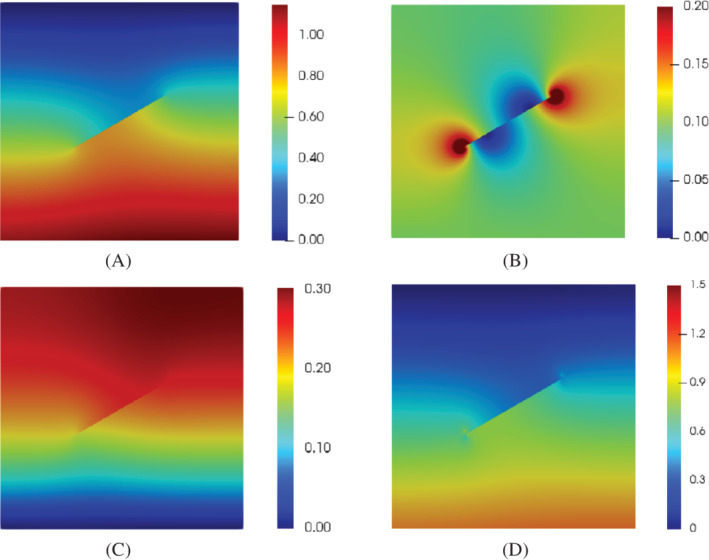
(A) Fluid pressure (MPa), (B) fluid flux norm $\left\|q\right\|$ (mm/s), (C) displacement norm (mm), and distribution of the (D) maximum principal stress (MPa) at time t=40s.

Figure 6 shows the pressure, the flux, the displacement and the stress distribution in the steady state $\left(t=40\;s\right)$. In Figure 6A, the fluid pressure is discontinuous across the crack. There is no flow across the interface. The fluid flows from one side of the interface to another side through the crack tips, illustrated in Figure 6B, which shows that the crack behaves like a barrier for the flow. Due to the impermeable crack interface, the displacement and the stress are also discontinuous across the crack. The pressure and displacement profile show a good agreement with the results reported by an interface element approach using Lagrange interpolations.^17^

### 5.2 Progressive fracturing in a single‐edge notched plate

**FIGURE 7:**
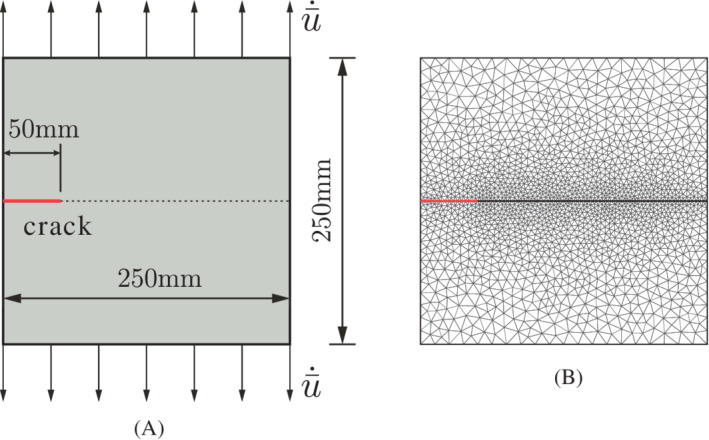
Square plate with an edge crack. The position of the crack is denoted by the red line. (A) Geometry and boundary conditions; (B) initial triangulation

A square plate with dimensions 250mm×250mm with an edge crack is now considered, see Figure 7. The length of the initial crack is a=50mm. We set the Young's modulus $25.85\times 10^{3}\;\text{MPa}$, the Poisson's ratio ν=0.18, the porosity $n_{f}=0.2$, and the intrinsic permeability $k=2.78\times 10^{-16}\;m^{2}$. The fluid has a viscosity $\mu =10^{-9}\;\text{MPa s}$. The bulk modulus of the solid is $K_{s}=13.46\times 10^{3}\;\text{MPa}$ and that of the fluid is $K_{f}=200\;\text{MPa}$. The Biot coefficient is α=1. The cohesive zone model in Equation (8) is used with the tensile strength $t_{u}=2.7\;\text{MPa}$ and fracture energy ${𝒢}_{c}=0.095\;\text{N/mm}$. We only consider mode‐I fracture, that is, $d_{\text{int}}=0$ in Equation (8). To avoid interpenetration, a penalty stiffness $k_{p}=10^{10}\;\text{MPa/mm}$ is specified in the normal direction of the crack opening. Vertical velocities $\dot{ū}=2.35\times 10^{-2}\;\text{mm/s}$ are applied at the top and the bottom sides of the plate. All boundaries are assumed to be impermeable, except for the crack interface for which the permeability, $k_{i}$, is set equal to the effective permeability coefficient of the porous medium $k_{f}$. The plate has been discretized by the triangulation shown in Figure 7B. The analysis is carried out with a time increment Δt=0.01s. Displacement control is employed to apply the velocity $\dot{ū}$ in the simulation, $\Delta u=\dot{ū}\times \Delta t=2.35\times 10^{-4}\;\text{mm}$. The displacement is imposed by the Lagrange multiplier method, compare Equation (21).

**FIGURE 8:**
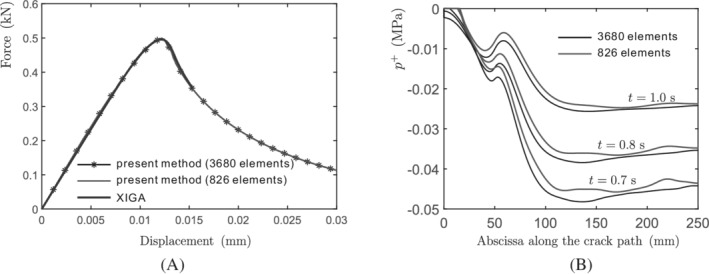
(A) Force‐displacement diagram and (B) pressure along the top crack interface $\Gamma_{c}^{+}$. In the figure, the results of two discretizations (3680 and 826 elements respectively) are shown

The computed load‐displacement curve is shown in Figure 8A with different number of elements. A good agreement is attained with the extended isogeometric analysis (XIGA),^8^ in which only the results, before the crack along the middle plane is fully opened, are shown. The curve exhibits structural softening due to progressive crack propagation. In the figure, a close agreement is observed between the two discretizations.^8^

**FIGURE 9:**
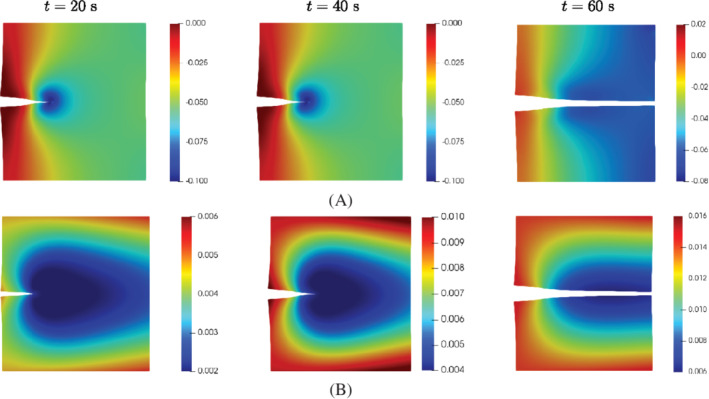
(A) Fluid pressure distribution (MPa) and (B) displacement norm (mm). Each column presents the results at time step t. The displacements have been amplified by a factor 1000.

Figure 8B presents the pressure $p^{+}$ along the top crack interface $\Gamma_{c}^{+}$ after the crack is fully developed along the middle plane of the plate. In addition, a comparison between the two discretizations is given for the fluid pressure $p^{+}$ here. A slight difference is observed in the figure due to the influence of mesh size. Obviously, the pressure acts as a tensile stress on the crack faces and shows a nonlinear distribution along the crack path, due to the nonlinear opening of the crack, see Figure 9B. The displacement of the plate increases with time, resulting in an increased crack opening, see Figure 9B. In the process of crack propagation, the pressure will concentrate around the crack tip (Figure 9A), due to the flow bypassing the crack tip. When the crack has fully opened, the fluid will gradually flow to the porous medium, progressively reducing the fluid pressure concentration, see the last picture in Figure 9A. Consequently, the pressure along the crack path gradually reduces to zero.

### 5.3 Arbitrary propagation: A plate with two propagating cracks

**FIGURE 10:**
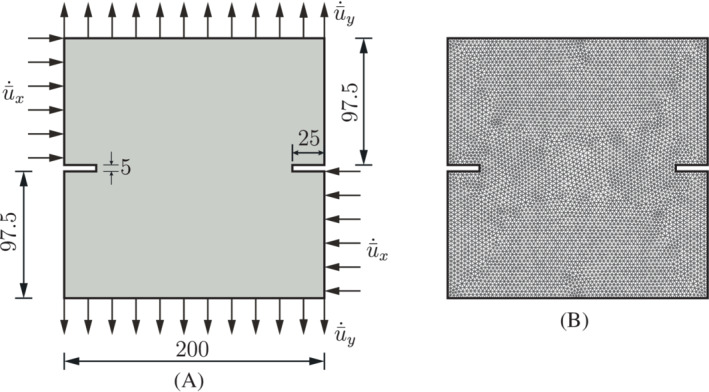
A plate with two propagating cracks. (A) Geometry and boundary conditions; (B) initial triangulation

This example serves to demonstrate the ability of the proposed method for mixed‐mode crack problems. The setup of the problem is similar to the Nooru‐Mohamed tension‐shear test.^44^ The Nooru‐Mohamed tension‐shear test has been carried out on a double‐edge notched plane concrete specimen with a thickness of 50 mm.^44^ Figure 10A shows the geometry and the boundary conditions. In the analysis, the specimen is first subjected to a prescribed horizontal velocity ${\dot{ū}}_{x}=2\times 10^{-2}\;\text{mm/s}$ until a certain level of horizontal displacement ${û}_{x}$ reached. Subsequently, a vertical velocity ${\dot{ū}}_{y}=2\times 10^{-2}\;\text{mm/s}$ was applied on the top and bottom edges while keeping ${û}_{x}$ constant. In the analysis, we consider the displacement ${û}_{x}=0.012\;\text{mm}$. The time increment is set as Δt=0.005s. A displacement control is employed to apply the velocity ${\dot{ū}}_{x}$ and ${\dot{ū}}_{y}$ in the simulation, $\Delta u_{x}={\dot{ū}}_{x}\times \Delta t=1.0\times 10^{-4}\;\text{mm}$ and $\Delta u_{y}={\dot{ū}}_{y}\times \Delta t=1.0\times 10^{-4}\;\text{mm}$. The displacement is imposed by the Lagrange multiplier method, Equation (21).

The material parameters are: Young's modulus E=30GPa, Poisson's ratio ν=0.2, Biot coefficient α=1, porosity $n_{f}=0.2$, intrinsic permeability $k=2.78\times 10^{-16}\;m^{2}$, solid bulk modulus $K_{s}=13.46\times 10^{3}\;\text{MPa}$, fluid bulk modulus $K_{f}=200\;\text{MPa}$, fluid viscosity $\mu =10^{-9}\;\text{MPa s}$. To investigate the influence of the interface permeability on the saturated porous medium, we take two different sets of values for the interface permeability: (1) $k_{i}=k_{f}$, and (2) $k_{i}=0$. To describe the fracture process, we employ the exponential decohesion relation of Equation (8) with a tensile strength $t_{u}=3.0\;\text{MPa}$ and a fracture energy ${𝒢}_{c}=0.11\;\text{N/mm}$. We now include mode‐II behavior: $d_{\text{int}}=10\;\text{N/mm}$ and $h_{s}=0$ in Equation (8).^45^ Plane‐stress conditions are assumed and the loading condition is set up as:

*Step* 1 Displacement control is considered to fully track the load‐displacement path with steps of $\Delta u_{x}$ along the upper left and bottom right edges. The top and bottom edges are fixed in the y‐direction at this stage, see Figure 10A. The total time is T=0.6s.
*Step* 2 Displacement control is employed to fully track the load‐displacement path with steps of $\Delta u_{y}$ along the top and bottom edges, while keeping ${û}_{x}=0.012\;\text{mm}$ constant along the upper left and bottom right edges, see Figure 10A.

**FIGURE 11:**
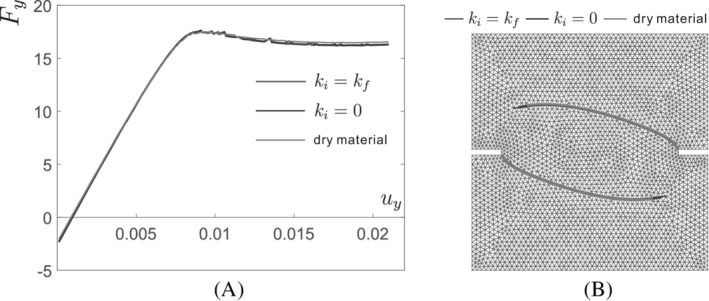
(A) Load (kN)‐displacement (mm) response and (B) predicted crack path

**FIGURE 12:**
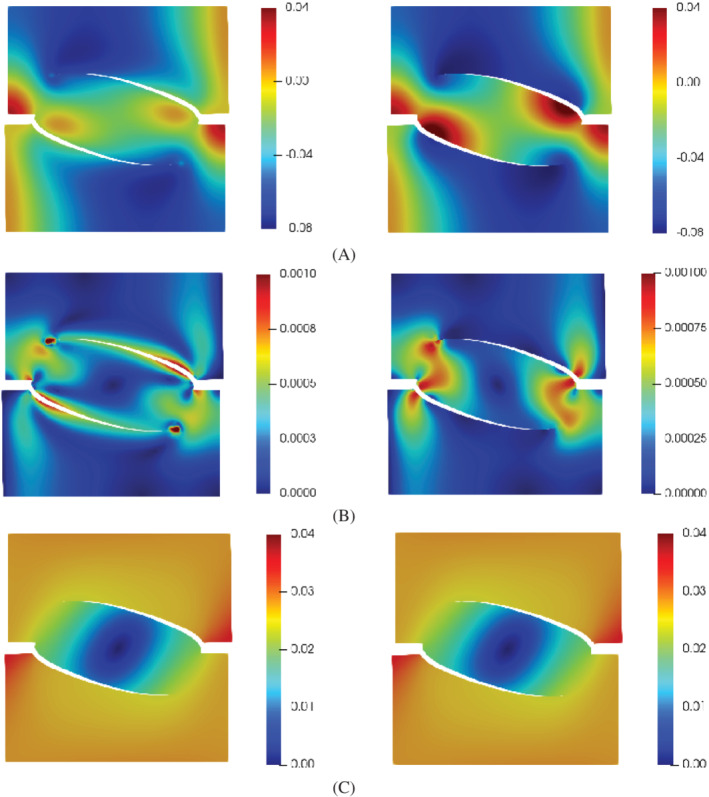
(A) Fluid pressure distribution (MPa), (B) fluid flux norm (mm/s), and (C) displacement norm (mm) for different settings of the interface permeability $k_{i}$. The left column presents the results of the interface permeability $k_{i}=k_{f}$, while the right column shows the results of $k_{i}=0$. The results at time step t=1.85s are shown. The displacements have been amplified by a factor 100.

The response curve is given in terms of the vertical resultant force $F_{y}$ versus the vertical displacement $u_{y}$ on the top edge at the loading Step 2, see Figure 11A. The parameter settings $k_{i}=k_{f}$ and $k_{i}=0$ give almost identical results, with a maximum difference of 1.67%, which indicates the fluid flow within the interface has a negligible influence on the response. In addition, we present the results in the case of dry material (no fluid) with identical set‐ups. Almost the same results are obtained for the cases of fluid‐saturated medium and dry material medium. This is also validated for the prediction of the crack path, given in Figure 11B. Obviously, the responses are dominated by the fracturing of the solid medium and the cohesive zone model. In the fracturing process, the crack opening gradually increases, which causes the decrease of the fluid pressure along the interface, as validated in Figure 8B. Consequently, the fluid pressure is considerably smaller than cohesive tractions, rendering a negligible influence on the responses in Figure 11. Figure 12 presents the fluid pressure, the flux, and the displacement distribution for the interface permeability $k_{i}=k_{f}$ and $k_{i}=0$, respectively. Obviously, different patterns are observed in the fluid pressure and flux profile. The fluid pressure difference $p^{+}-p^{-}$ and the flux difference $q^{+}-q^{-}$ along the crack interface, in the case of $k_{i}=k_{f}$, are smaller than those in the case of $k_{i}=0$, as illustrated in Figure 12A,B. This is caused by the impermeable interface set‐up in the case of $k_{i}=0$. For the displacement profile, both settings of the interface permeability $k_{i}$ induce almost identical patterns, as presented in Figure 12C.

## 6 CONCLUSIONS

A Powell‐Sabin finite element scheme has been developed for progressive fracturing in a fluid‐saturated medium. A cohesive zone law governs the interface behavior of the solid part, while a two‐pressure degrees of freedom (2PDOF) model manages the fluid flow within the crack. ${𝒞}^{1}$‐continuous Powell‐Sabin B‐splines, which are based on triangles, are employed for the geometry, the displacement and the fluid pressure approximation. Due to the ${𝒞}^{1}$ continuity property, one can directly assess the crack initiation at the crack tip.

In the process of crack propagation, the crack is directly introduced in the physical domain, different from the discrete modeling in the isogeometric analysis framework. Due to the use of triangular elements, remeshing is straightforward. After remeshing new Powell‐Sabin B‐spline functions are introduced, which requires a mapping of the state vector (displacement and fluid pressure) from the old to the new mesh. A novel least‐squares fit methodology has been proposed to carry out the mapping. To preserve the energy and mass in the transfer, the energy balance and mass conservation are chosen as constraints.

Numerical examples are presented to validate the proposed method and to show the refinement ability of the Powell‐Sabin B‐splines. For the chosen examples, the interface permeability plays a minor influence on the response, except for the fluid pressure and the flux. This is due to the fact that the fluid pressure decreases upon an increase of the crack opening. Consequently, the fracture behavior of the solid and the cohesive zone law control the crack propagation.

Due to certain constraints with neighboring tetrahedrons, the extension of Powell‐Sabin B‐splines to three‐dimensional objects is non‐trivial,^46^ but one can construct prisms as a tensor product of two‐dimensional Powell‐Sabin B‐splines and NURBS in the third dimension.

In the analysis, we employed the same basis functions for the interpolation of the displacement field and the pressure field, which does not comply with the LBB or *inf‐sup* condition.^47^ Nevertheless no oscillations in the pressure field were observed, which may be due to the higher‐order continuity of the Powell‐Sabin B‐splines.

## CONFLICT OF INTEREST

The authors declare that there is no conflict of interest regarding the publication of this article.

## Data Availability

Data sharing is not applicable to this article as no datasets were generated or analyzed during this study.
